# Patella-Femoral Joint Revision Surgery Following a Rare and Interesting Case of Implant Failure: A Case Report and Retrospective Analysis of Our Hospitals Implant Data

**DOI:** 10.7759/cureus.22908

**Published:** 2022-03-07

**Authors:** Joshua K Nadimi, Daniel Winson, William Roy, Daniel W Lewis, David Pemberton

**Affiliations:** 1 Trauma and Orthopaedics, University Hospital of Wales, Cardiff, GBR; 2 Trauma and Orthopaedics, University Hospital of Llandough, Cardiff, GBR; 3 Trauma and Orthopaedics, Royal Glamorgan Hospital, Llantrisant, GBR

**Keywords:** : patellofemoral, knee, arthroplasty, implant failure, revision, femoro-patella vialla

## Abstract

According to the national joint registry, patello-femoral joint (PFJ) replacement accounts for 1% of all knee arthroplasty procedures in the UK, 1014 of which were performed in 2018. The femoro-patella vialla (FPV) implant by MicroPort orthopaedics has a high reported rate of revision, more than four times that of other knee replacements.

The mechanisms of failure are usually loss of fixation at the bone-implant interface. We observe a rare and unusual case form of patella component failure whereby the facets of the implant remain imbedded within the patella as a result of shear force, leaving a loose patella button within the knee.

A literature review was conducted of reported modes of failure of the FPV implant and retrospective analysis of our units’ results looking into rate of revision, modes of failure, and assessment of a single surgeon’s patient outcome measure (Knee Society Scores).

Retrospective analysis of our hospital's data revealed that 11% of FPV implants were revised in our unit, a large percentage of which were due to pain and progressive osteoarthritis. We observed revision rates of 5.84% at 3 years, 10.21% at 5 years, and 13.13% at 10 years, which is favourable to national joint registry revision estimates of 6.99%, 10.13%, and 19.10%, respectively.

Knee Society Scores reveal a mean improvement of 39.65 (−38 to 100) at follow-up following FPV unicompartmental knee arthroplasty surgery. Our unit demonstrated better results from patient outcome measures when compared to literature and a lower rate of revision when compared to national figures.

## Introduction

According to the national joint registry [1], patellofemoral joint (PFJ) replacement accounts for 1% of all knee arthroplasty procedures in the UK, 1,646 of which were performed in 2019. Unicompartmental arthroplasty for the PFJ offers a good option for the younger patient with isolated anterior compartment arthritis as opposed to total knee arthroplasty [2].

The "femoro-patella vialla (FPV)" implant by MicroPort orthopaedics (Wright Medical, Memphis) was designed by Professor Vialla in France over 20 years ago [3]. The trochlear implant has a broad angle of 140 degrees and a longer, steeper lateral slope compared to the medial (similar to the nature of a true, anatomical trochlea). There is a 90-degree curve in the sagittal plane, designed to prevent dislocation of the patella in deep flexion [3,4]. The patellar component is an asymmetrical, facetted implant with an oval footprint that slopes medially from proximal to distal. These design considerations are to replicate the normal anatomy and kinematics of the knee [4].

The revision rate for patellofemoral joint replacement is 5.6 times higher than total knee replacements [1]. In a systematic review of patellofemoral arthroplasty failures, pain was found to be the most common reason for early failure, and late failure was attributed most commonly to progressive tibio-femoral arthritis [5]. There are studies that demonstrate high early revision rates of the FPV implant, but none of these were related to component failure [6,7].

Our case demonstrates an unusual form of patella component failure whereby the implant facets of the implant remain imbedded within the patella as a result of shear force, leaving a loose patella button within the knee.

## Case presentation

**Figure 1:**
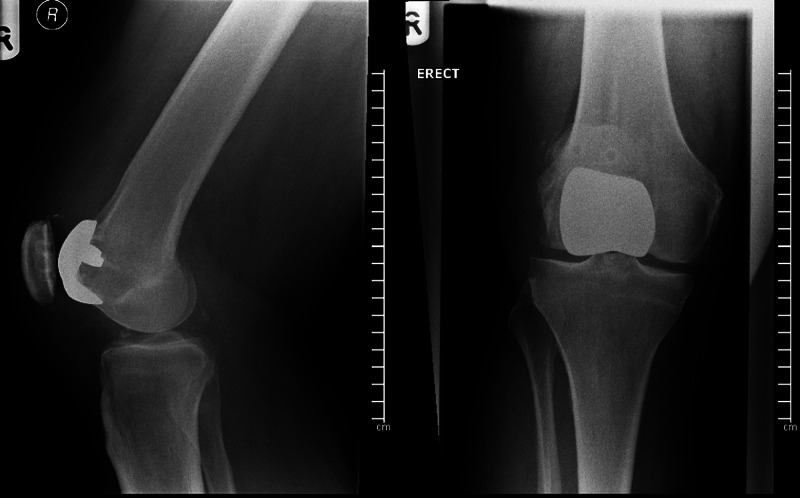
Post-operative radiographs of right knee following primary femoro patella vialla arthroplasty. Antero-posterior (right) and lateral (left) non-weight bearing radiographs of the right knee following primary patellofemoral arthroplasty. The femoral FPV component is well positioned on the trochlea. The patella button is correctly positioned in the patella and congruent with the femoral implant component.

A 68-year-old mechanical engineer with isolated patellofemoral joint arthritis and a past medical history of gout, osteoarthritis, and gastroesophageal reflux disease underwent bilateral femoro-patella vialla joint arthroplasty (Wright Medical) in 2009 (Figure 1).

**Figure 2:**
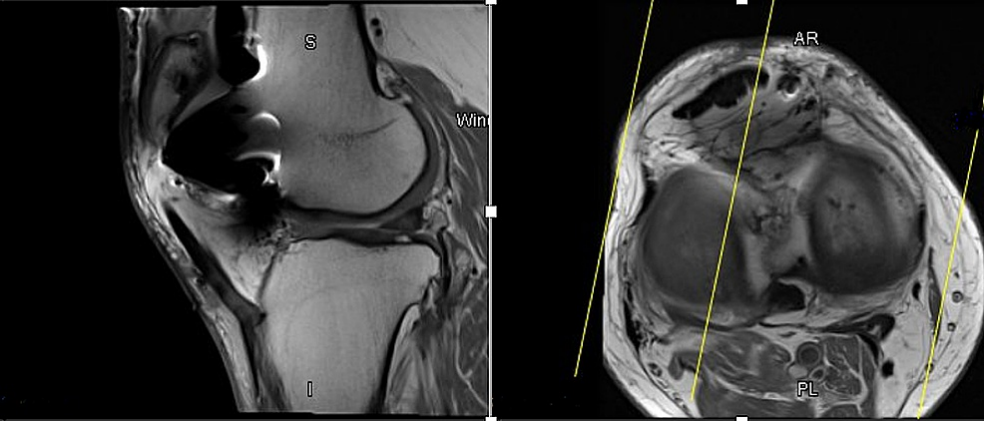
Magnetic resonance images of the right knee displaying lateral meniscus tear. The left image is a sagittal proton density sequence slice of the right knee in the lateral compartment displaying a lateral meniscal tear and artefact from the patellofemoral implant. The right image is a proton density fat suppressed sequence axial slice at the level of the tibial plateau displaying a lateral meniscal tear.

This gentleman was satisfied with his surgery, and his short-term post-operative follow-up was unremarkable. Ten years following the primary surgery, the patient was walking up a hill when he had a sudden onset pain in his anterior right knee, associated with a large effusion 24 hours later. Investigations were negative for infection, and plain AP and lateral radiographs of the affected knee revealed no new bony pathology or implant loosening. Magnetic resonance imaging later revealed a lateral meniscus tear but no demonstrated abnormality in the implant (Figure 2).

**Figure 3:**
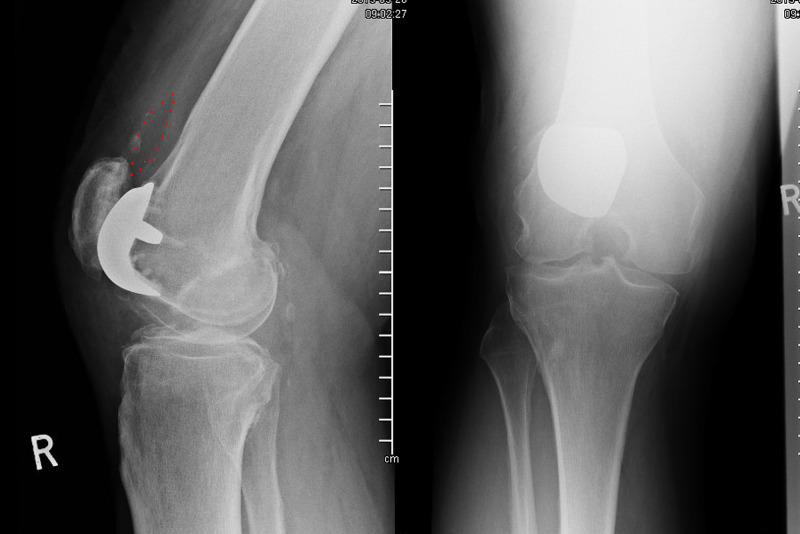
Pre-operative radiographs of the right knee displaying loose patella button (outlined on lateral view). Anteroposterior (right) and lateral (left) non-weight bearing radiographs of the right knee. The patella is lying directly on the femoral component of the implant and outlined in red is the shadow of the dislocated patella button of the failed FPV arthroplasty, lying within the suprapatellar pouch.

An arthroscopy was performed for debridement of the meniscal tear and intraoperatively the implant appeared well fixed. The patient continued to complain of persistent anterior knee pain and recurrent effusions within the right knee. Radiographs following the initial arthroscopy were suspicious for failure and displacement of the patella button (Figure 3).

**Figure 4:**
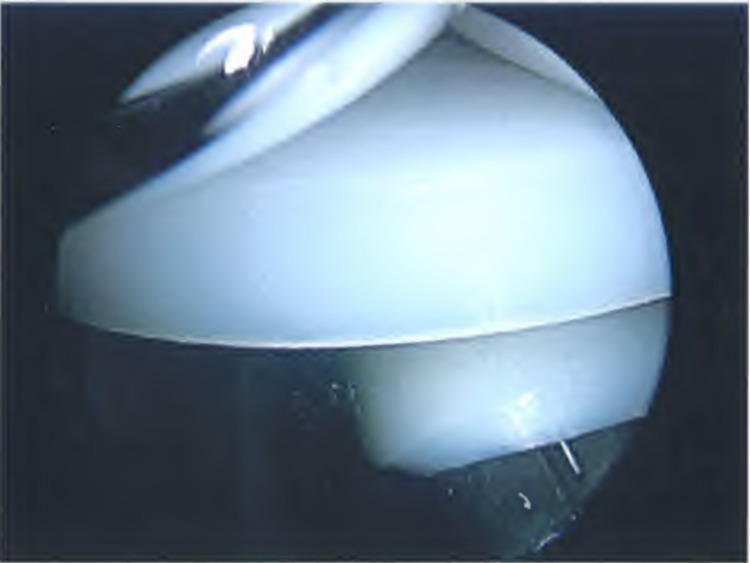
Arthroscopic image of displaced patella component. Image taken on arthroscopy of the right knee displaying the FPV implant and displaced patella component loose within the suprapatella pouch.

A second arthroscopy was performed revealing that the patella button was loose within the suprapatellar pouch (Figure 4) and the decision was made to proceed to revision surgery by open arthrotomy.

**Figure 5:**
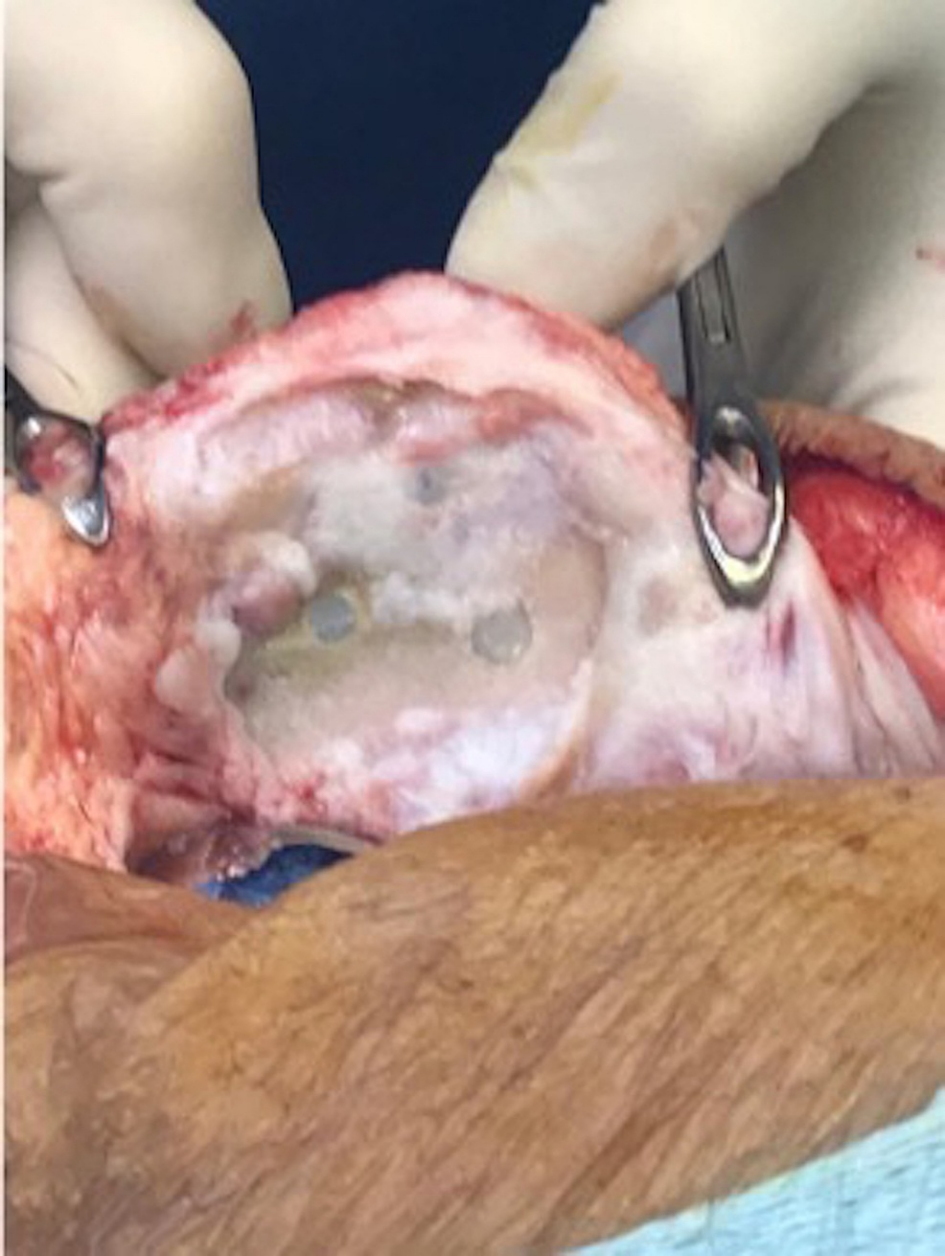
Intraoperative images during resurfacing procedure for a failed FPV patellofemoral implant. The intraoperative image displays the underside of the everted right patella during an open arthrotomy for patella resurfacing procedure. The three retained "pegs" from the failed patella button are visualised within the body of the patella.

An open arthrotomy of the right knee was performed, exposing the patellofemoral compartment. Intraoperatively, the failed patella button was found to have sheared from the base, leaving the pegs still within the patella (Figure 5). The femoral component remained well fixed and stable.

**Figure 6:**
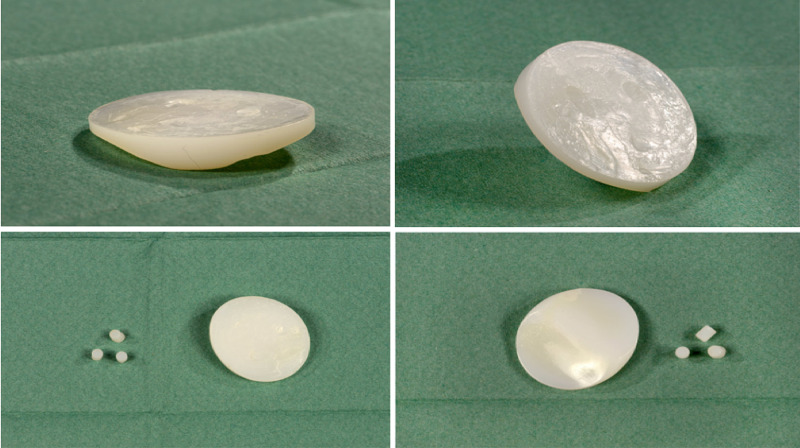
Photographs of the failed implant displaying separate implant base and pegs. Four clinical photographs show the failed patella button component of an Femoro-Patella Vialla implant. The top two images show the patella component and footprints of the three broken "pegs." The bottom two images show the patella component and the retrieved "pegs" from the patients patella.

Figure 6 displays the failed patella implant and the sheared "pegs" from the base of the device.

**Figure 7:**
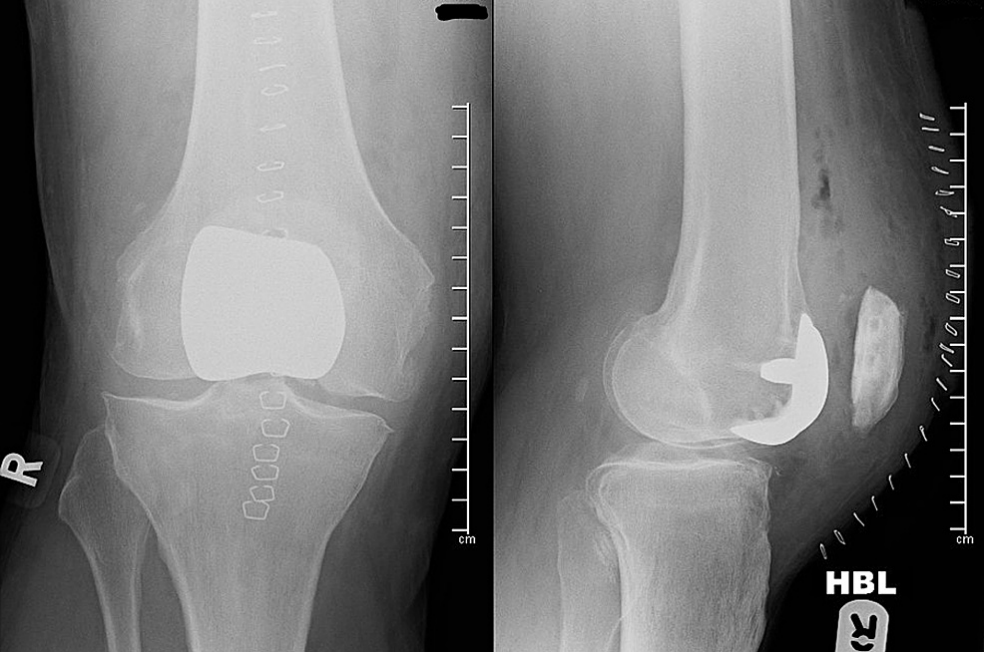
Post-operative radiographs of the right knee following revision of the patella component. The left image is an anteroposterior radiograph of the right knee and the left image a lateral radiograph of the same (non-weight bearing). The patellofemoral joint is congruent following patella revision to an "Onlay Poly" (Microport Orthopaedics) and skin clips are visible over the anterior aspect of the knee.

The patella component was successfully revised to an "Onlay All-Poly" patella implant (Microport Orthopaedics, Inc., Arlington, TN). Patella tracking and on-table examination was stable in addition to satisfactory post-operative radiographs and a good post-operative outcome (Figure 7).

## Discussion

Revision rates for patella-femoral joint arthroplasty are significantly higher than for other knee replacements, and total knee replacement surgery remains the most popular surgical option for the treatment of osteoarthritis of the knee [1].

The UK National Joint Registry's top three reported failure modes of patellofemoral arthroplasty include osteoarthritis progression, pain, and "other" [1]. Further reasons for revision include surgical error, wear, infection, stiffness, fracture, and broken patella component. A recent review of patellofemoral arthroplasty revisions by van der List et al. concurs with progressive osteoarthritis and pain as the most common modes of implant failure and reports aseptic loosening reported at a rate of 14% [8]. Interestingly, Davies reports that in all cases of FPV patellofemoral replacements that were revised, the patella button was poorly fixed in all cases [7]. A separate systematic review of patellofemoral arthroplasty failure modes across a number of implants identified only 2% of PFA failures have been attributed to broken patella components and none with the Femoro-Patella Vialla implant specifically [9].

It seems the most common mode of failure for the patella implant component specifically is the pull-out of facets from the bone (aseptic loosening). To the best of our knowledge, there is no reported mode of failure in the Femoro-Patella Vialla implant whereby the facets of the patella shear away from the base of the implant (as shown in Figures 5-6).

Our patient was a suitable candidate for patella-femoral arthroplasty with isolated patellofemoral disease and no contraindicated anatomical anomalies or intraoperative complications. This case must therefore be attributed to a rare form of patella component failure which, to the best of our knowledge, has not been previously reported.

The FPV has a faceted trochlea and patella component in addition to a proximally extended lateral trochlea groove, which allows for an increased contact area and earlier engagement of the patella component to the trochlea. In their biomechanical analysis of the implant, Monk et al. demonstrated significant differences between the FPV implant and normal knee kinematics at mid-flexion, which included cantilevering of the patella at the end of the trochlea component [4]. We postulate that repeated loading of the implant in mid-flexion may have caused repeated and increased shear forces across the patella bone-implant interface. This mechanism, in addition to fatigue wear, may have resulted in this rare mode of failure. Further analysis would be required to investigate the biomechanical factors relating to this mode of failure in order to test our theory.

We retrospectively analysed 137 patients from our unit who received FPV patellofemoral arthroplasty in the 2007-2019 period. 13% of FPV implants were revised, and the primary reasons for failure and subsequent revision were pain (4%) and osteoarthritis progression (4%). These are consistent with commonly reported modes of failure in the literature and according to the national joint registry. We observed three cases of failure secondary to aseptic patella component loosening, which were all revised to alternate patellofemoral joint prostheses.

National Joint Registry data from 2019 states that 1,646 femoro-patella vialla prostheses were implanted in the UK. Kaplan-Meier estimates for the cumulative percentage probability of FPV prostheses first revision were 6.99%, 10.13%, and 19.10% at 3 years, 5 years, and 10 years, respectively [1]. Following retrospective analysis of FPV prostheses implanted in our unit, we observed revision rates of 5.84% at 3 years, 10.21% at 5 years, and 13.13% at 10 years, which is favourable to National Joint Registry revision estimates.

Furthermore, Knee Society Scores collected from our FPV patient dataset pre-operatively and one-year post-operatively reveal a mean improvement of 46.60 at follow-up, indicating a good overall outcome for our patients.

## Conclusions

We display an interesting form of femoro-patella vialla implant failure whereby the facets of the patella component of the implant remain imbedded within the patella, creating a loose body within the knee. As far as we are aware, this is a rare mechanism of failure and the only documented failure of its kind. Surgeons should be aware of such modes of failure not only specific to the femoro-patella vialla implant, but all PFJ arthroplasty devices with facet-containing patella components.

Furthermore, a retrospective review of our unit's FPV arthroplasty patients demonstrated a lower rate of revision of the device at 3 and 10 years when compared to national joint registry data and a mean improvement in post-operative Knee Society scores.
